# Pulmonary valve neocuspidization and tricuspid valve replacement in intravenous drug abusers with infective endocarditis: Report of two cases

**DOI:** 10.1093/icvts/ivac063

**Published:** 2022-03-16

**Authors:** Borys Todurov, Igor Mokryk, Bohdan Batsak, Nataliya Ponych

**Affiliations:** Department of Adult Cardiac Surgery, Heart Institute, Kyiv, Ukraine

**Keywords:** Infective endocarditis, Ozaki, pulmonary valve neocuspidization

## Abstract

Right-sided infective endocarditis accounts for 5–10% of endocarditis cases. It occurs predominantly among intravenous drug abusers. The pulmonary valve is involved in fewer than 2% of patients with endocarditis. Literature data are limited and optimal medical strategy, including surgical technique, remains non-standardized in this clinical situation. We present 2 patients treated surgically for tricuspid and pulmonary valve endocarditis and discuss a method of pulmonary valve neocuspidization based on the Ozaki technique.

## INTRODUCTION

The incidence of right-sided infective endocarditis is growing mainly due to the increasing numbers of intravenous drug abusers (IVDA), the rising number of implantable cardiac devices and prolonged use of central venous catheters [1]. Despite the proven effectiveness of intravenous antibiotic therapy, from 5–40% of these patients have to undergo surgery [2]. Literature on the operative management of pulmonary valve (PV) infective endocarditis (IE) is minimal [2–4]. The optimal surgical technique is under debate. Ozaki reported promising mid-term results of the aortic valve neocuspidization method [5]. We used this principle for PV neocuspidization (PVNeo) in 2 patients with right-sided infective endocarditis.

## PATIENTS

Both patients were young males (patient 1 was 39 and patient 2 was 21 years old) with a history of IVDA and hepatitis C. Despite previous long-term optimal medical therapy, including intravenous antibiotics, their condition progressively worsened, and they were referred to our hospital. Upon admission, they complained of debilitation, high fever and shortness of breath on physical exertion. Patient 1 had cachexia (body mass index 15.6) and hydroperitoneum on observation. A chest X-ray and a computed tomography scan demonstrated severe two-sided destructive pneumonia in patient 1 and left-sided pneumonia with hydrothorax in patient 2. Blood cultures revealed *Staphylococcus* *aureus* and *Escherichia* *coli* in patient 1 and *S.* *aureus* in patient 2. Transthoracic echocardiography demonstrated massive (more than 1 cm) vegetations and insufficiency of the tricuspid valve (TV) and PV in both cases. Patients were qualified for surgery because of ineffective previous medical therapy, severe haemodynamic compromise, large vegetations and septic embolization to the lungs.

## SURGICAL TECHNIQUE

**Figure 1:**
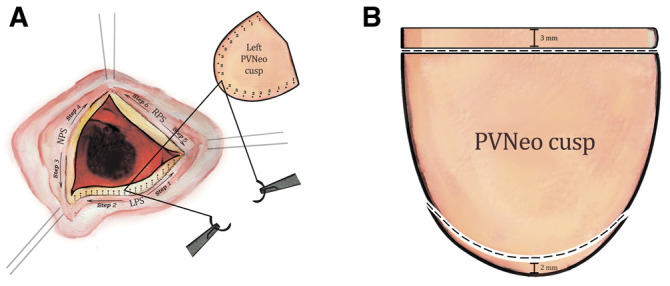
Schematic illustration of a pulmonary valve neocuspidization (**A**) The sequence of leaflet replacement and the bite-to-bite ratio is indicated. (**B**) Schematic illustration of a pulmonary valve neocuspidization cusp trimming to correspond to the remaining pulmonary valve leaflets in patient 1. LPS: left pulmonary sinus; NPS: non-facing pulmonary sinus; RPS: right pulmonary sinus; PVNeo: pulmonary valve neocuspidization).

After a midline sternotomy, a 7- × 8 -cm piece of autopericardium was harvested and treated with 0.625% glutaraldehyde solution for 10 min, followed by triple rinsing in saline for 6 min each time. Because of severe destruction, the TV was replaced with a bioprosthesis in both cases. Transverse pulmonary arteriotomy 1 cm above the level of the commissures was used to approach the PV. In patient 2, all 3 cusps were damaged. Video 1 demonstrates annotated guidance of the operative technique in this patient. Cusps of the PV were excised. The intercommissural distance was measured, and the corresponding leaflets (two 25 -mm and one 23 -mm) were trimmed using sizers and a template developed by Ozaki [5]. The leaflets were fixed to the corresponding annulus using a 4.0 polypropylene running suture in the following sequence: left, non-facing and right. A smooth pericardial surface should face the right ventricle to avoid thrombocytopaenia. Suture starts at the nadir and travels towards the top of the commissure. New leaflets are larger than native ones; therefore, significant plication should be performed. Bigger bites are taken on the patch (ratio 3:1) while suturing near the nadir. More equal bites (2:1 and 1:1) are taken while approaching the commissures (Fig. 1A). At the top of the commissures, sutures are brought outside of the PA and fixed on pledgets. The final alignment of leaflets was achieved by applying a vertical 5.0 polypropylene suture at the top of commissures.

In patient 1, only the right cusp had to be replaced. It measured 23 mm and was implanted as described above. To correspond to the remaining native leaflets, the height of the PVNeo cusp was made 5 mm shorter by cutting a 3 -mm strip from the top and a 2 -mm semilunar strip from the bottom of the cusp (Fig. 1B).

**Figure 2.**
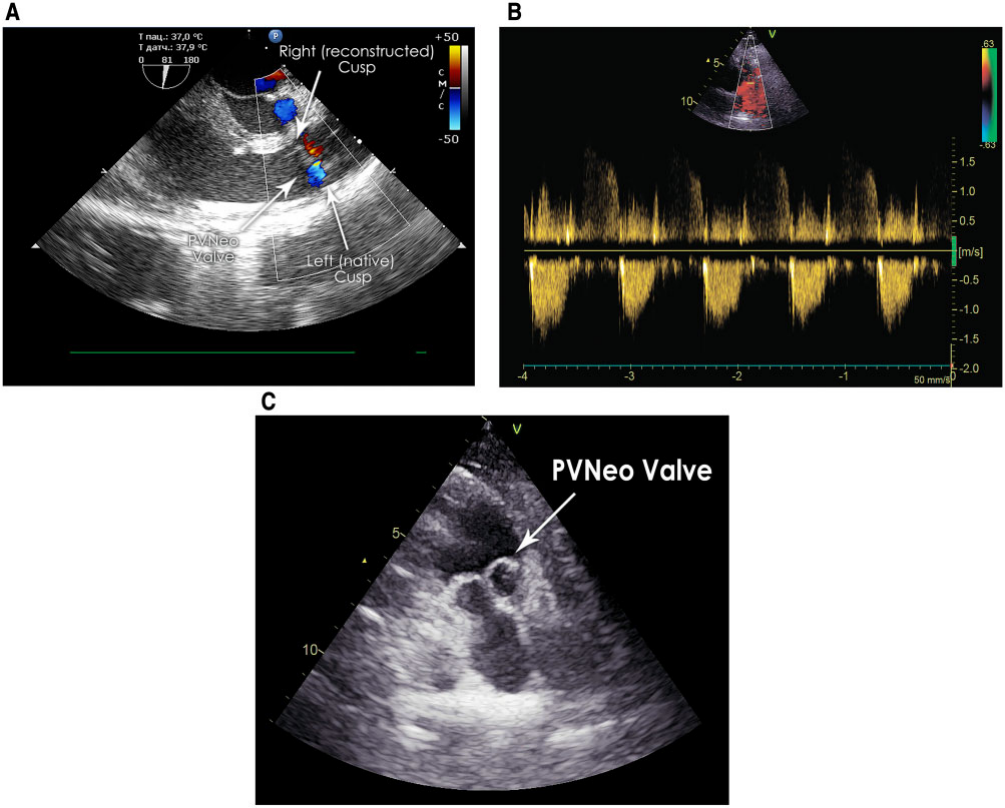
(**A**) Intraoperative transoesophageal echocardiography demonstrating good geometry and function of the reconstructed pulmonary valve in patient 1. (**B**) Colour Doppler image showing good haemodynamic performance of the same pulmonary valve neocuspidization after 2 years. (**C**) Transthoracic echocardiography (modified short-axis view) demonstrating good geometry and function of the pulmonary valve neocuspidization in patient 2 before discharge. PVNeo: pulmonary valve neocuspidization.

Intraoperative transoesophageal echocardiography demonstrated excellent PVNeo valve geometry and function in both cases (Fig. 2A).

## RESULTS

The postoperative period of patient 1 was complicated by respiratory insufficiency due to previous destructive pneumonia. He was discharged on postoperative day 59. At the 2-year follow-up, the patient is in good condition. On echocardiography, we noted a TV bioprosthesis with trace insufficiency, Δρ 10/5 mmHg and a PVNeo valve with trace insufficiency, Δρ 9/4 mmHg (Fig. 2B).

The in-hospital course of patient 2 was uneventful, and he was discharged on postoperative day 13. His transthoracic echocardiographic scan confirmed excellent PVNeo valve geometry and function before discharge (Fig. 2C). The patient was readmitted 9 months postoperatively due to repeat endocarditis associated with continuous drug abuse. Transoesophageal echocardiography revealed vegetations on the TV bioprosthesis while the PVNeo valve was intact. The patient refused redo surgery and was discharged on medical therapy.

For prophylaxis of PVNeo valve thrombosis, both patients were given warfarin for 1 month postoperatively and then were given low-dose aspirin for 6 months.

## COMMENT

Right-sided infective endocarditis responds well to optimal medical therapy. Surgery is reserved for patients with poor reaction to treatment, persistent bacteremia, severe haemodynamic compromise, large vegetations and septic embolization to the lungs.

The behaviour of glutaraldehyde-treated autologous pericardium used for reconstruction of the aortic valve is comparable to that of the biological prostheses at the midterm follow-up [5, 6]. The Ozaki technique has proven its effectiveness in the treatment of aortic valve pathology with the longest follow-up period of up to 10 and a half years [5]. Current studies demonstrate minimal differences in the geometry of the aortic and pulmonary roots [7]. They served as a scientific basis to apply the Ozaki concept for PV reconstruction. Only several publications report such experiences to date [3, 4, 8]. They all demonstrate good immediate outcomes. We present our experience with PVNeo and an excellent up to 2 years follow-up result.

Even despite a good immediate outcome, the risk of reoperation in the IVDA group is high [1]. This outcome is mainly due to the relapse of intravenous drug use and consequent recurrent endocarditis. A subgroup of patients, after planned addiction treatment and proper social support, overcome the risk of intravenous reinfection. They will come at the mid- and long-term follow-up period at a still young age. They should be closely monitored for possible structural valve deterioration just like any young patient with a biological valve prosthesis [9]. Therefore, in patients after PVNeo, all efforts should be made to secure safe eventual sternal re-entry. A large pericardial defect should be reconstructed with proper, commercially available synthetic or biological material. Meticulous haemostasis, complete pericardial retained blood drainage, aggressive prophylaxis of postoperative wound infection and mediastinitis will help prevent the development of massive dense adhesions.

The PVNeo technique is flexible, and, if necessary, only 1 or 2 destroyed cusps of the native valve may be replaced, as was done in patient 1 [4]. Materials are readily available, and the method is economically attractive.

PVNeo is exposed to lower pressure stress than the original Ozaki valve in the aortic position. If a more delicate PA wall and annulus are not severely damaged with the infective process, no additional interrupted sutures are needed to ensure the long-term competence of a continuous suture line.

PVNeo does not have a rigid stent. It preserves the natural mobility of the right ventricle outflow tract and results in a better haemodynamic outcome [5, 9]. This valve may be implanted in a very small annulus without the need for enlargement of the right ventricle outflow tract [4, 8]. Also, the method avoids the placement of any synthetic material into the bloodstream. These factors may be the potential advantages of PVNeo over PV replacement with bioprostheses in terms of better haemodynamic performance, the safety of the procedure and resistance to repeat endocarditis [10]. However, a more significant number of patients is required to prove this concept.

**Conflict of interest**: none declared.

## Notes

Meeting presentation: The 35th EACTS Annual Meeting, Barcelona, 15 October 2021

## References

[1] Wang A, Gaca J, Chu V. Management considerations in Infective Endocarditis. A Review. JAMA 2018;320:72–83. 10.1001/jama.2018.7596 29971402

[2] Shmueli H, Thomas F, Flint N, Setia G, Janjic A, Siegel RJ. Right-sided infective endocarditis 2020: challenges and updates in diagnosis and treatment. J Am Heart Assoc 2020;9:e017293. 10.1161/JAHA.120.017293 32700630 PMC7792231

[3] Jaswal V, Thingnam S, Kumar V, Munirathinam G. Surgical valve repair of native pulmonary valve endocarditis using Ozaki technique. Ann Thorac Surg 2020;110:e509–11. 32445631 10.1016/j.athoracsur.2020.03.125

[4] Kumar R, Halder V, Gourav KP, Patel R, Munirathimnam GK, Mandal B et al Pulmonary valve neocuspidization with glutaraldehyde-treated autologous pericardium – A novel technique in pulmonary valve endocarditis. J Card Surg 2020;35:1725–8. 10.1111/jocs.14659 32579761

[5] Ozaki S, Kawase I, Yamashita H, Uchida S, Takatoh M, Kiyohara N. Midterm outcomes after aortic valve neocuspidization with glutaraldehyde-treated autologous pericardium. J Thorac Cardiovasc Surg 2018;155:2739–87. 10.1016/j.jtcvs.2018.01.087 29567131

[6] Al Halees Z, Shahid M, Al Sanei A, Sallehuddin A, Duran C. Up to 16 years follow-up of aortic valve reconstruction with pericardium: a stentless, readily available cheap valve? Eur J Cardiothorac Surg 2005;28:200–5. 16039963 10.1016/j.ejcts.2005.04.041

[7] Berdajs D, Zund G, Schurr U, Camenisch C, Turina M, Genoni M. Geometric models of the aortic and pulmonary roots: suggestions for the Ross procedure. Eur J Cardiothorac Surg 2007;31:31–5. 17126557 10.1016/j.ejcts.2006.10.037

[8] Takahashi Y, Shibata T, Fujii H, Morisaki A, Sakon Y, Yamane K et al Aortic and pulmonary valve reconstruction using autologous pericardium in narrow annuli. Ann Thorac Surg 2020;109:t13–5. 10.1016/j.athoracsur.2019.04.070 31185205

[9] Cocomello L, Meloni M, Rapetto F, Baquedano M, Ordoñez MV, Biglino G et al Long-term comparison between pulmonary homografts versus bioprosthesis for pulmonary valve replacement in tetralogy of Fallot. Jaha 2019;8:e013654. 10.1161/JAHA.119.013654 31838974 PMC6951084

[10] Ali-Ghosh H, Barlow CW. Commentary: bioprosthetic pulmonary valve endocarditis: another “arrow in our quiver”. JTCVS Tech 2021;6:73–4. 34318148 10.1016/j.xjtc.2021.01.039 PMC8300971

